# Assessment of chitosan nanoemulsion loading with perillaldehyde on the quality characteristics and microbial diversity of beef slices during refrigeration storage

**DOI:** 10.1016/j.fochx.2025.102658

**Published:** 2025-06-12

**Authors:** Shengming Zhao, Jingyao Wu, Mengke Li, Yanyan Zhao, Guoyuan Xiong, Xinkun Wang, Lizeng Peng

**Affiliations:** a School of Food Science and Engineering, Anhui Science and Technology University, No.9 Donghua Road, Fengyang 233100, PR China; b Key Laboratory of Novel Food Resources Processing / Institute of Agro-Food Sciences and Technology, Shandong Academy of Agricultural Sciences, Jinan 250100, China; c School of Food Science and Technology, Henan Institute of Science and Technology, No.90 Hua lan Street, Xinxiang 453003, PR China; d Anhui Province Key Laboratory of Functional Agriculture and Functional Food, Anhui Science and Technology University, Chuzhou 239000, PR China

**Keywords:** Beef, Chitosan, Nanoemulsion, Perillaldehyde, Storage

## Abstract

This study investigated the effect of a chitosan nanoemulsion enriched with perillaldehyde on the preservation quality and microbial community composition of fresh refrigerated beef. After 15 days of storage, the perillaldehyde incorporation in the chitosan nanoemulsions (PA-CS) group exhibited the lowest pH (6.61), cooking loss (34.88 %), thiobarbituric acid reactive substances (TBARS, 0.86 mg MDA/kg), and total carbonyl content (1.47 nmol/mg protein). The total volatile base nitrogen (TVB-N) and total viable counts (TVC) of the PA-CS group on day 15 were 15.85 mg/100 g and 5.69 CFU/g, respectively, which just slightly exceeding the regulatory standards for fresh meat. The PA-CS group effectively maintained color stability, slowed the texture deterioration, and decreased the transformation of immobilized water to free water in refrigerated beef. Furthermore, high-throughput sequencing indicated that PA-CS coating significantly affected the bacterial community composition. In conclusion, chitosan nanoemulsions incorporated with perillaldehyde are a promising method for beef preservation.

## Highlights

• Chitosan nanoemulsions loading with perillaldehyde was used for beef preservation.
• The nanoemulsions decreased lipid and protein oxidation in beef.
• The nanoemulsions improved the texture and water distribution of beef.
• The nanoemulsions altered the microbial diversity of refrigerated beef.

## 1 Introduction

Beef, one of the most widely consumed meats globally, is rich in essential nutrients, including high-quality proteins, healthy fats, essential minerals, and various vitamins, and has a distinctive flavor (Li et al., 2022). Recently, the consumption foods have rapidly increased worldwide, driven by economic development and improved living standards in several countries. Moreover, China is among the fastest-growing countries in terms of beef imports and consumption (Ansarian et al., 2022; Cao et al., 2022). Fresh beef is typically transported and stored under refrigeration at temperatures between 0 and 5 °C. However, spoilage and pathogenic microorganisms in fresh beef can still proliferate under refrigeration, leading to gradual nutrient degradation caused by enzymatic activity (Zhu, Gu, et al., 2024; Zhang, Wang, et al., 2023). Owing to its high nutrient content, fresh beef is highly susceptible to oxidative deterioration and microbial contamination during transportation, storage, and sale, which is an increasing concern for consumers. The spoilage of fresh beef negatively affects its physicochemical properties and sensory attributes, such as texture, color, and odor, ultimately leading to reduced consumer acceptance and economic losses for manufacturers (Yan et al., 2024). Therefore, developing novel, effective, and convenient preservation techniques to extend the shelf life of fresh beef while maintaining its quality has become a pressing issue in the food industry.

Recently, plant essential oils, characterized by their antibacterial and antioxidant activities, have been considered natural and safe food preservatives to that prolong food shelf life (Isvand et al., 2024; Zhang et al., 2024). Perillaldehyde, a major hydrophobic active compound found in the essential oil extracted from perilla plants (*Perilla frutescens*), has garnered widespread interest due to its numerous biological activities, including antimicrobial properties and various human health benefits. Historically, it has been extensively used in traditional Chinese medicine as a food additive and flavoring agent (Erhunmwunsee et al., 2022; Masumoto et al., 2019). It can be used as a natural food preservative to replace synthetic chemical preservatives by inhibiting microbial growth through the disruption of the integrity of cell walls and membranes (Zhu, Zou, et al., 2024). However, its poor water solubility and oxidative instability limit its application in the food industry. Chitosan nanoemulsions, are an emerging eco-friendly technology that has been increasingly utilized in food processing and preservation (Chaudhary et al., 2022). Chitosan nanoemulsions are widely recognized as effective delivery vehicles for hydrophobic substances owing to their simple preparation process, small particle size, enhanced solubility, and controlled release of active compounds. These properties enhance the interactions between active compounds and cell membranes, thereby inhibiting microbial proliferation and improving antioxidant activity. (Ansarian et al., 2022; Rathod et al., 2024; Zhang et al., 2024). Furthermore, chitosan nanoemulsions can act as a protective barrier, preventing moisture evaporation of moisture and oxygen permeation during short-term food storage (Zhang et al., 2020). Consequently, they have been used as alternative methods to enhance meat product and delay microbial spoilage (Prasad et al., 2022; Wang, Liu, et al., 2022).

Fresh beef is vulnerable to microbial contamination, lipid and protein oxidative deterioration during storage because of its rich nutrient composition, including moisture, carbohydrates, and unsaturated lipids (Zhu, Gu, et al., 2024). The undesirable growth and metabolism of microorganisms are the primary cause for meat product spoilage during storage (Cao et al., 2023). The spoilage bacteria can cause lipid oxidation and protein degradation by producing lipolytic and proteolytic enzymes, resulting beef spoilage and shelf life reduction (Xu et al., 2024). Understanding the changes in bacterial community during beef refrigeration can contribute to exploit quality control and preservation strategies for refrigerated meat. Xu et al. (2024) found that vacuum skin packaging reduced the microbial diversity and improved the oxidation stability of beef during refrigerated storage. Jiang et al. (2024) reported that hydrogel coatings composed of konjac glucomannan*, Lactiplantibacillus plantarum* and gallic acid could significantly change microbial community and effectively reduced the abundance of *Pseudomonas*, leading to the reduced lipid oxidation and prolonged storage of beef. Inhibiting microbial growth and enhancing beef during storage through the application of nanoemulsions loaded with natural active compounds has been recognized as a promising strategy. To the best of our knowledge, no study has explored the effects of chitosan nanoemulsion loaded with perillaldehyde on microbial diversity and shelf-life of fresh refrigerated beef. Accordingly, the motivation for the present study was to investigate the effect of this treatment on the quality attributes of beef during 15 days of refrigerated storage by evaluating changes in pH, cooking loss, color, thiobarbituric acid reactive substances (TBARS), carbonyls, carbonyl content, total volatile base nitrogen (TVB-N), texture, water distribution, sensory characteristics, total viable counts (TVC) and bacterial diversity.

## 2 Materials and methods

### 2.1 Materials and chemicals

Fresh bovine tenderloin (Xianan cattle, approximately 20 months old, pH 5.83 ± 0.07, moisture 69.53 ± 0.67 %, protein 22.13 ± 0.38 %, fat 2.64 ± 0.14 %, initial TVC 3.79 ± 0.08 lg CFU/g, aged for 24 h after humane slaughter under cooling conditions) was obtained from a local supermarket (Xinxiang, China). Chitosan (161.16 kDa, 90 % deacetylation) was obtained from Aladdin Chemical Co. (Shanghai, China). Perillaldehyde (purity, ≥ 92 %) was acquired from Yuanye Biological Technology Co., Ltd. (Shanghai, China). Gelatin was supplied by Yinuo Biotechnology Co., Ltd. (Quzhou, China). Tween 80, and thiobarbituric acid (TBA) were procured from Kemio Co., Ltd. (Tianjin, China).

### 2.2 Preparation of the chitosan nanoemulsion

Chitosan nanoemulsion was prepared according to the methods described by Huang et al. (2020) with slight modifications. The chitosan-gelatin solution containing 0.5 % chitosan (*w*/*v*) dissolved in acetic acid and 2 % (w/v) gelatin solution, was mixed for 2 h at 25 °C using a magnetic stirrer (LCS-UMS-6, Lichen Corporation, Shanghai, China) at 120 rpm. The pH of the solution was adjusted to approximately 4.0 using 5 mol/L NaOH. Then, 1 mL of the perillaldehyde was mixed with 100 mL of the chitosan-gelatin solution and 4 mL of Tween 80, then homogenized at 25 °C using a homogenizer at 10,000 rpm (T-25 Ultra Turrax, IKA, Germany) until a uniform emulsion premix was obtained. Finally, the chitosan nanoemulsion loaded with perillaldehyde was processed using a high-pressure homogenizer at 30 MPa for five cycles.

### 2.3 Preparation of the chitosan nanoemulsion loaded with perillaldehyde on beef meat

Fresh beef samples were obtained from a local supermarket and transported to the laboratory in polystyrene boxes containing ice (Xinxiang, China). After detaching the fat and connective tissue, the beef samples were sliced into small pieces (3 × 3 × 1 cm). Subsequently, all samples were rinsed with ultrapure sterilized water and were divided into the following three experimental groups: (1) The chitosan nanoemulsion treated (CS) group consisted of beef samples immersed in a chitosan nanoemulsion for 1 min; (2) The chitosan nanoemulsion with perillaldehyde treated (PA-CS) group included beef samples immersed in a chitosan nanoemulsion with perillaldehyde for 1 min; (3) the control (CK) group consisted of beef samples that were untreated with chitosan nanoemulsion or chitosan nanoemulsion with perillaldehyde. All samples were then placed in aseptic polyethylene trays and stored at 4 °C for 15 days. Three samples from each treatment group were analyzed for various quality parameters on day 0, 3, 6, 9, 12, and 15 (Wang, Sun, et al., 2022).

### 2.4 pH

The pH was determined as described by Ansarian et al. (2022). A 5 g meat sample was finely chopped and blended with 25 mL of potassium chloride solution (0.75 g/L). After homogenization at 10,000 rpm for 30 s, the pH of the meat sample was measured using a digital pH meter (Seven Compact S220; Mettler, Zurich, Switzerland).

### 2.5 Cooking loss

The vacuum-packed samples (20 g) were heated at 85 °C for 20 min until the core temperature reached 75 °C. The samples were then chilled to 25 °C, and the surface moisture was removed by blotting paper. Finally, the samples were weighed (Cao et al., 2022). Cooking loss was calculated using the following formula:

Cooking loss (%) = 100 × (W_0_-W_1_)/W_0_ (1).

Where W_0_ is the weight (g) of the sample before cooking, and W_1_ is the weight (g) of the sample after cooking.

### 2.6 Instrumental color

The color change in the meat samples were measured using a chroma meter CR-400 (Konica Minolta, Tokyo, Japan) (Wang et al., 2022a). The *L*^*⁎*^(lightness)、*a*^*⁎*^(redness) and *b*^*⁎*^(yellowness) values were recorded for each sample A standard white plate was used for calibration (*L*^*⁎*^ = 96.83, *a*^*⁎*^ = −0.75, *b*^*⁎*^ = 1.57). Each portion of the meat sample was subjected to five measurements each.

### 2.7 TBARS

The TBARS values of meat samples were measured according to Zhang, Liu, et al. (2023). Briefly, chopped meat (5 g) was blended with 50 mL of a 7.5 % trichloroacetic acid solution for 1 min and incubated at 50 °C for 30 min. Afterward, the mixtures were filtered, and 5 mL TBA solution was mixed with 5 mL of the collected filtrate and subjected to a reaction at 95 °C for 40 min. The reaction products were centrifuged (4 °C, 3000 ×*g*) for 10 min (L-80-XP, Beckman, USA) and the supernatant was used to read the absorbance at 532 nm using a UV-1800 spectrophotometer (Shimadzu Corporation, Kyoto, Japan). The TBARS values were extrapolated from a standard malondialdehyde (MDA) response curve (y = 1.0105× + 0.0094) and expressed as mg MDA/kg.

### 2.8 Total carbonyls

Meat samples (1 g) were blended thoroughly with 10 mL of 0.15 M KCl using a homogenizer at 10,000 rpm for 60 s. The two obtained suspensions (0.5 mL) were mixed with 0.5 mL of trichloroacetic acid (10 %). After centrifugation for 5 min at 5000 ×*g*, one precipitate was treated with 1 mL of 2 M HCl containing 0.2 % 2,4-dinitrophenyl hydrazine (DNPH) to determine carbonyl content, while 2 M HCl (1 mL) was added to another precipitate to determine the protein concentration. Each sample was then mixed with 1 mL of 10 % trichloroacetic acid. After centrifugation at 5000 *g* for 5 min, the precipitates were retrieved and washed thrice with 1 mL of ethanol/ethyl acetate (1,1). Subsequently, the precipitates were mixed with 1.5 mL of 20 mM sodium phosphate buffer containing 6 M guanidine hydrochloride. After centrifugation for 5 min at 5000 ×*g*, the supernatants were assayed for the absorbance of carbonyls and protein concentration at 370 and 280 nm, respectively. The protein concentration was extrapolated from a standard bovine serum albumin response curve (Ansarian et al., 2022). The carbonyl content was calculated using the following formula:

(2) $$\text{Carbonyl content}\;\left(\text{nmol}/\mathrm{mg}\;\text{protein}\right)=100\times \frac{{\mathrm{Abs}}_{370\mathrm{nm}}}{21.0\;{\mathrm{mM}}^{-1}\;{\mathrm{cm}}^{-1}}$$

where 21.0 mM^−1^ cm^−1^ is the molar extinction coefficient of carbonyls in nmol/mg of protein.

### 2.9 TVB-N

The quantification of TVB-N content adhered to the procedures outlined by Lu et al. (2019). Briefly, each homogenized beef sample (20 g) was extracted with 100 mL of trichloroacetic acid and shaken 30 min to ensure complete protein extraction. Following filtration, 5 mL of the filtrate, 5 mL of a 10 g/L MgO solution, and 2 drops of dimethicone were added to a Kjeldahl distillation apparatus. The mixture was distilled for 5 min, and the distillate was collected in a 10 mL of boric acid solution (20 g/L). Subsequently, the distillate was titrated with 0.01 mol/L hydrochloric acid. The TVB-N content was evaluated based on the consumption of hydrochloric acid and calculated using the following formula:

(3) $$\mathrm{TVB}-N\;\left(\mathrm{mg}/100\;g\right)=100\times \frac{\left(V1-V2\right)\times c\times 14}{m}$$

where m is the weight of the meat sample (g), c is the concentration of the HCl standard titration solution (mol /L), V1 is the titration volume of HCl for the meat sample (mL), V2 is the titration volume of HCl for the blank (mL), and 14 is the molecular weight of nitrogen.

### 2.10 Texture

The texture of the meat samples was analyzed using a texture analyzer (TA-XT plus, Stable Micro Systems, Surrey, UK), as described by Wang et al. (2024). For texture profile analysis, the meat sample was cooked at 85 °C for 20 min. Then, after equilibrating to room temperature (20 °C) for 2 h, the samples were cut into a cylinder (φ2.8 cm × 20 mm). The TPA mode and P/36 R probe were used to analyze the hardness, springiness, cohesiveness, and chewiness. The experimental parameters included a compression ratio of 40 %, test speed of 1 mm/s, pre-test and post-test speeds of 2 mm/s each, a trigger force of 5 g, and an interval duration of 5 s.

### 2.11 TVC

The meat samples (25 g) were homogenized with 225 mL of sterile saline solution (0.09 g/kg NaCl) for 2 min using a flapping homogenizer (Scientz-09, Ning Bo Scientz Biotechnology Co. Ltd., China). Then the uniform mixture was diluted using sterile saline solution by ten-fold serials. 1 mL of each sample solution was dispensed onto plate count agar (Aobox Co., Ltd., Beijing, China) using a 10-fold dilution, followed by incubation at 37 °C for 48 h for TVC analysis. All operations were performed on a sterile operating table. Data are presented as log_10_ CFU/g.

### 2.12 Low-field nuclear magnetic resonance (LF-NMR)

The meat samples were uniformly cut into cubical pieces with dimensions of 1.5 cm × 1.5 cm × 2.0 cm and then positioned within a low-field NMR glass tube for further measurements using a LF-NMR analyzer (Model PQ001, Niumag Electric Corporation, Shanghai, China) at a resonance frequency of 22.6 MHz. The CPMG sequence was used to quantify the spin-spin relaxation time (T_2_) and the associated water cluster distribution ratio (PT_2_) with a τ value of 350 μs. 10,000 echoes were acquired by 32 scans of the samples with a repeat interval of 2.5 s. The T_2_ values were collected by inversion analyses with Multi-ExpInv NMR software (Zhao et al., 2024).

### 2.13 Sensory evaluation

Sensory evaluation was performed by 15 trained postgraduate students (8 female and 7 male, aged on average 24.54 ± 1.5, range 23–27 years) majoring in food science before they were included in this study. They assessed four samples in a single session for each storage time and conducted a total of six sessions for each replication. The assessment criteria included color, odor, texture, and overall acceptability using a 9-point hedonic scale (1, extremely dislike; 9, extremely like) (Cao et al., 2022). The experimental procedure was approved by the Institutional Review Board (School of Food Science and Engineering, Anhui Science and Technology University), All panelists in the sensory experiments were informed of the experimental procedure and voluntarily agreed to participate in the study. Each panelist provided consent to participate in the experiment and to have their data used as part of the study. The obtained sensory data was conducted by a univariate analysis of variance. In this analysis, treatments and panelists were incorporated as fixed factors, while sessions were considered as a random variable nested within storage time.

### 2.14 DNA extraction and sequencing analysis

The DNA was extracted from the microbial communities using the method described by Wang et al. (2022a). DNA was extracted from each sample using a bacterial DNA extraction kit (OMEGA BioTek). The universal primers 341F (5′-CCTACGGGNGGCWGCAG-3′) and 805R (5′-GACTACHVGGGTATCTAATCC-3′) were used to amplify the bacterial V3-V4 variable regions of 16S rDNA. The PCR amplicons were characterized by 2 % agarose gel electrophoresis, followed by the recycling and purification of the desired DNA fragments using the Quant-iT PicoGreen dsDNA Assay Kit. Three identical DNA samples from the same treated group were pooled together, and the DNA concentration was quantified employing a NanoDrop 2000 UV–Vis spectrophotometer (Thermo Scientific, Waltham, USA). Illumina libraries were constructed using the TruSeq™ Nano DNA LT Library Prep Kit (Illumina, lnc., USA). Sequencing was performed using an Illumina NovaSeq 6000 sequencer (APTBIO Co., Ltd., Shanghai, China). The PE reads obtained from sequencing were subjected to quality control, including filtering, denoising, splicing, and dechimerization to obtain amplicon sequence variants with optimized sequences and abundance data. Subsequently, bacterial community diversity analysis was performed using the QIIME software (version 2.0).

### 2.15 Statistical analysis

Data analysis was conducted using the SPSS v.26.0 (SPSS Inc., Chicago, USA). The results are presented as the mean ± standard deviation (SD). A one-way analysis of variance (ANOVA) followed by Duncan's multiple range test was employed to determine statistical significance, with *P* < 0.05 considered significant. Each experimental measurement was performed independently on three occasions, and four different levels were assessed in each of the trials.

## 3 Results and discussion

### 3.1 Changes in pH values

**Fig. 1:**
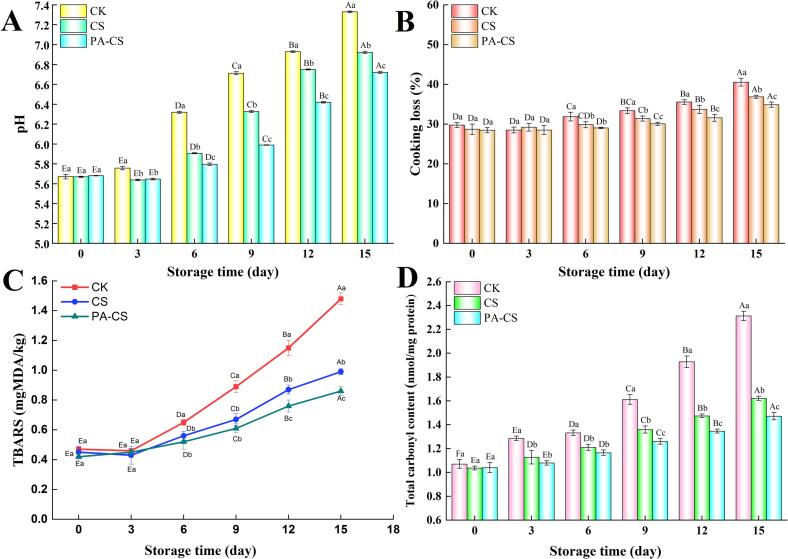
Enumeration of pH (A), cooking loss (B), TBARS (C), and total carbonyl content (D) of beef with and without treatment during refrigeration. Different capital letters (A-F) indicate significant differences within the same batch (*P* < 0.05); different lowercase letters (a-c) indicate significant differences within the same sampling time (*P* < 0.05).

The metabolism of spoilage microorganisms in fresh meat during refrigeration can increase the pH, value, thereby affecting the storage quality of fresh meat (Zhang, Liu, et al., 2023). Therefore, pH determination is a vital indicator for assessing the quality attributes of fresh meat. The variations in the pH of beef during refrigeration are depicted in Fig. 1A. The pH values of CK and different nanoemulsion treatment groups continuously increased throughout refrigeration as the storage time progressed (*P* < 0.05). This trend was consistent with previously reported findings on meat storage with coating treatment (Snoussi et al., 2022; Wang et al., 2022a; Zhang, Liu, et al., 2023). This phenomenon can be explained by the fact that alkaline compounds, such as ammonia and amines are progressively generated and accumulated due to the combined effects of intrinsic enzymes and microbial proliferation, which degrade proteins as the storage time increases. This process increases the pH of meat samples. (Zhang, Liu, et al., 2023). However, the pH values of different chitosan nanoemulsion treatment groups exhibited a significantly slower increase than those of the CK group, and the PA-CS group demonstrated the lowest pH value throughout the refrigeration period. On the 15th day, the pH values in CK, CS, and PA-CS groups increased to 6.93, 6.72, and 6.61, respectively, indicating that the different chitosan nanoemulsion treatments could inhibit the growth of microorganism in beef during refrigeration, contributing to the slowing of the increase in pH value. Comparable findings have been reported by Dini et al. (2020) and Wang et al. (2022a), who found that chitosan nanoemulsion loaded with cumin essential oil and chitosan nanoemulsion with thymol decreased the pH values of beef and pork during cold storage. Owning to the antibacterial activities of perillaldehyde and chitosan, the growth of spoilage microorganisms that produce volatile basic nitrogen compounds was inhibited, resulting in a slower rate of pH increase in the beef sample (Wang et al., 2022b). Furthermore, the barrier effect against air contact with the beef surface also inhibited the growth of microorganisms, which is another important factor.

### 3.2 Change in cooking loss

The acceptability of beef is influenced by cooking loss, which indicates a reduction in intramuscular fluids, including water and soluble nutrients such as certain amino acids and nucleotides. The variations in the cooking loss of beef during refrigeration are displayed in Fig. 1B. The cooking loss of the CK and different nanoemulsion treatment groups gradually increased (*P* < 0.05) with the prolonged storage duration. Protein oxidation, induced by meat endogenous enzymes and microbial metabolites, disrupts the muscle fiber microstructure and results in the leakage of the water and nutrients (Bellucci et al., 2022). During the 15 days storage period, the different nanoemulsion treatment groups maintained a lower cooking loss than the CK group, and the PA-CS group demonstrated the lowest cooking loss throughout refrigeration. At the end of storage on the 15th day, the cooking loss in CS and PA-CS groups was reduced by 9.12 % and 13.98 %, respectively, compared to the CK group. The lowest cooking loss in PA-CS group could be attributed to the antibacterial and antioxidant activities of perillaldehyde and chitosan. Perillaldehyde and chitosan inhibited microbial propagation and decelerated the decomposition of nutrient components in beef, contributing to the reduction in cooking loss. Additionally, the lower protein oxidation in the PA-CS group further explained the decreased cooking loss, which was consistent with the findings on carbonyls (Fig. 1D) (Cao et al., 2022). Comparable results were reported by Ghani et al. (2018), who found that cinnamon essential oil nanoemulsions prevented greater weight loss in beef during 8 days of storage at 4 °C. Therefore, the application of chitosan nanoemulsion with perillaldehyde can effectively delay the increase in beef cooking loss during refrigeration by inhibiting microbial spoilage and protein oxidation.

### 3.3 Change in instrumental color

As presented in Table 1, the different nanoemulsion treatment groups showed a higher *L*^*⁎*^ value than the CK group in the initial stage of refrigeration, which could be attributed to the chitosan nanoemulsion coating on the beef surface. Additionally, *L*^*⁎*^ and *a*^*⁎*^ values of CK and different nanoemulsion treatment groups exhibited a declining trend (*P* < 0.05) with increasing storage duration. This could be explained by the fact that myoglobin reacted with oxygen to form greater amounts of high iron myoglobin over time, resulting in a decrease in *L*^*⁎*^ and *a*^*⁎*^ values. Moreover, the rapid growth of spoilage bacteria in beef with increasing storage time accelerates myoglobin oxidation, leading to the production of metmyoglobin (Cao et al., 2022). Throughout the storage period, the nanoemulsion treatment groups displayed remarkably higher *L*^*⁎*^ and *a*^*⁎*^ values compared to the CK group, and the PA-CS group maintained the peak value. This is attributed to the fact that the antioxidant activities of chitosan and perillaldehyde can retard beef discoloration by complexing with Fe^2+^ ions (Cao et al., 2022). Furthermore, the excellent antibacterial properties of chitosan and perillaldehyde were key factors contributing to the stability of *L*^*⁎*^ and *a*^*⁎*^ values by inhibiting microbial growth (Wang et al., 2022a). The *b*^*⁎*^ values of the CK group significantly increased from 12.12 to 18.15 after 15 days of storage, whereas the *b*^*⁎*^ values of CS and PA-CS groups increased from 11.65 to 16.23 and from 11.26 to 14.85, respectively. The increased *b*^*⁎*^ values of beef throughout the storage period might be due to the interaction involving lipid oxidation products and amines, whereas chitosan and perillaldehyde might exert protective effects against lipid oxidation, resulting in a reduced formation of lipid oxidation products, which is consistent with the TBARS results (Fig. C). Dghais et al. (2023) showed that cinnamon nanoemulsion could effectively slow down beef discoloration by inhibiting the emergence of methemoglobin and lipid oxidation. Sutharsan et al. (2023) reported that chitosan films with flavonoid treatment exhibited higher color stability in beef compared to the CK group during chilled storage. Overall, the chitosan nanoemulsion loaded with perillaldehyde effectively retarded beef discoloration during storage.

**Table 1:** Enumeration of color of beef with or without treatment during refrigeration.

	Storage time (days)					
	0	3	6	9	12	15
*L*^*⁎*^						
CK	44.77 ± 1.21^Ab^	40.54 ± 0.71^Bb^	36.19 ± 1.40^Cb^	35.81 ± 0.56^Cc^	33.27 ± 0.48^Dc^	31.13 ± 0.34^Ec^
CS	48.71 ± 0.73^Aa^	44.96 ± 1.87^Ba^	42.61 ± 0.78^Ba^	40.44 ± 0.47^Cb^	37.75 ± 0.26^Db^	35.98 ± 0.57^Eb^
PA-CS	49.49 ± 1.07^Aa^	45.88 ± 0.96^Ba^	43.83 ± 1.24^Ba^	42.26 ± 0.26^Ba^	41.26 ± 0.56^Ca^	38.02 ± 0.47^Da^
*a*^*⁎*^						
CK	21.39 ± 0.68^Aa^	18.06 ± 0.61^Ba^	11.06 ± 0.52^Cb^	7.52 ± 1.04^Dc^	5.12 ± 0.48^Eb^	3.91 ± 0.85^Fc^
CS	20.41 ± 0.95^Aa^	18.44 ± 0.53^Ba^	17.05 ± 1.05^Ba^	13.6 ± 0.76^Cab^	9.56 ± 0.32^Db^	7.54 ± 0.11^Eb^
PA-CS	20.74 ± 1.42^Aa^	17.74 ± 0.45^Ba^	17.99 ± 0.53^Ba^	15.59 ± 0.48^Ca^	12.81 ± 0.94^Da^	9.61 ± 0.49^Ea^
*b*^*⁎*^						
CK	12.12 ± 0.68^Da^	12.55 ± 0.7^Da^	13.81 ± 0.31^Ca^	16.82 ± 0.59^Ba^	16.22 ± 0.42^Ba^	18.45 ± 0.43^Aa^
CS	11.65 ± 0.51^Da^	11.43 ± 0.56^Da^	11.78 ± 0.8^CDb^	12.38 ± 0.44^Cb^	13.72 ± 0.29^Bb^	16.23 ± 0.46^Ab^
PA-CS	11.26 ± 0.87^Ca^	11.67 ± 0.53^Ca^	11.46 ± 0.49^Cb^	11.41 ± 0.28^Cc^	12.67 ± 0.53^Bb^	14.85 ± 0.22^Ac^

Note: Values with different capital letter letters (A-F) in the same batch were significantly different (P < 0.05); Values with different lowercase letters (a-c) at the same sampling time were significantly different (P < 0.05).

### 3.4 Change in TBARS

TBARS, which primarily originate from the oxidative degradation of phospholipids and unsaturated fatty acids, are commonly used to assess lipid oxidation rancidity, a process that generates off-odors during meat storage (Ansarian et al., 2022). The variations in the TBARS values of beef during refrigeration are depicted in Fig. 1C. On day 0 of refrigerated storage, the initial TBARS values of CK, CS, and PA-CS groups were 0.47, 0.45, and 0.42 mg MDA/kg, respectively, and gradual increase was observed in all treatments during the 15 days storage. The increased TBARS value in beef was attributed to the oxidative degradation of polyunsaturated fatty acids in beef, which was triggered by the oxygen penetration (Yan et al., 2024). Degradation products, such as glutaraldehyde and hexanal, can could cause offensive odors. Moreover, lipid oxidation can promote the oxidation of myoglobin and heme iron, contributing to a darker color (Zhang, Liu, et al., 2023), which coincided with the decrease in the *L*^*⁎*^ value (Table 1). However, the CK group exhibited the fastest increasing rate in TBARS values throughout the entire storage period, and CK, CS, and PA-CS groups reached peak values of 1.48, 0.99, and 0.86 mg MDA/kg on the 15th day, respectively. Specifically, the PA-CS group exhibited distinctly lower TBARS levels than other groups (*P* < 0.05). This may be attributed to the strong antioxidant properties of perillaldehyde and chitosan, such as their ability to decompose peroxides and scavenge free radicals, which inhibited the oxidation of unsaturated fatty acids in the beef. Additionally, the robust oxygen barrier effect of the edible coatings formed by the chitosan nanoemulsion contributed to the inhibition of lipid oxidation (Sun et al., 2021). Nanoemulsions containing natural plant extracts have been applied to different types of meat to reduce TBARS levels in meat (Ansarian et al., 2022; Snoussi et al., 2022; Sun et al., 2021). Snoussi et al. (2022) reported a similar finding, demonstrating that a nanoemulsion loaded with Tunisian thyme essential oil effectively decelerated the increase in TBARS values of fresh bovine meat during storage at 4 °C.

### 3.5 Change in total carbonyl

The oxidative degradation of specific amino acid side chains, including lysine, proline, arginine, and histidine, leads to the formation of carbonyl compounds, which are frequently used to assess the extent of protein oxidation (Amiri et al., 2019). The total carbonyl content of all groups increased during the storage period (Fig. 1D). Notably, increased carbonyl levels in meat indicate exacerbated oxidative protein damage. Moreover, the occurrence of protein oxidation in meat is closely tied to lipid oxidation, as the primary and secondary products of lipid oxidation can interact with amino acid residues via covalent bonds, initiating indirect protein oxidation processes (Lund et al., 2011), as further evidenced by the TBARS results (Fig. 1C). However, the increase in carbonyl content in CS and PA-CS groups was significantly lower than that in the CK group throughout the storage period (*P* < 0.05), with peak values of 1.62, 1.47 and 2.31 nmol/mg protein at the end of the storage period (day 15), respectively. This reduction was attributed to the antioxidant activities of chitosan and perillaldehyde, which protect against protein oxidation. Perillaldehyde enhances the antioxidant ability of beef by activating antioxidant enzymes such as superoxide dismutase and catalase (Erhunmwunsee et al., 2022). Similarly, a chitosan film incorporating cumin essential oil nanoemulsion has been reported to decrease carbonyl levels in beef during refrigerated storage (Dini et al., 2020). Ansarian et al. (2022) also reported that nanoemulsions containing resveratrol and clove essential oil significantly reduced total carbonyl contents in camel meat compared with the CK group during chilled storage.

### 3.6 Change in TVB-N

**Fig. 2:**
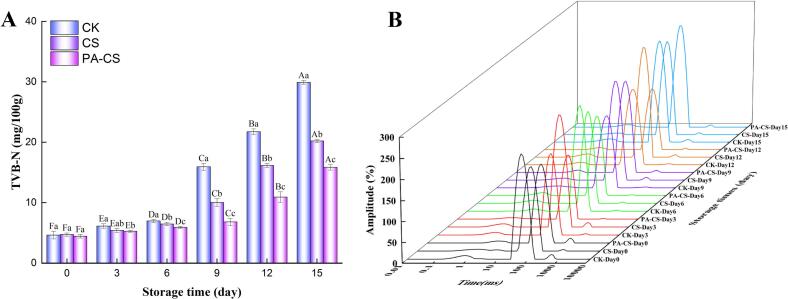
Enumeration of TVB-N (A) and distribution of T_2_ relaxation times (B) of beef with and without treatment during refrigeration. Different capital letters (A-F) indicate significant differences within the same batch (*P* < 0.05); different lowercase letters (a-c) indicate significant differences within the same sampling time (*P* < 0.05).

The denaturation of proteins in meat, induced by endogenous enzymes and bacteria under refrigerated conditions, leads to the generation of basic nitrogenous compounds (TVB-N), such as ammonia and amines, which are frequently used as crucial indicators for assessing meat freshness (Wang et al., 2022a). According to Chinese Standard GB 2707–2016, the permissible TVB-N limit for fresh livestock products is a maximum of 15 mg/100 g. As can be seen in Fig. 2A, the TVB-N values of all beef samples gradually increased over time, and all groups were < 7 mg/100 g during the initial 6 day of storage, indicating good quality preservation. However, after 6 days, the TVB-N values of the CK group increased sharply, exceeding the regulatory limit of 15 mg/100 g on day 9 (15.94 mg/100 g). A possible explanation is that the accelerated growth and proliferation of spoilage bacteria leads to the protein degradation, contributing to the production of abundant volatile basic nitrogenous substances, such as ammonia and trimethylamine. Another important factor is the continuous accumulation of ammonia and trimethylamine, which increases the pH to a level favorable for endogenous cathepsin activity, thereby promoting rapid protein decomposition (Zhang, Liu, et al., 2023). However, CS and PA-CS groups exhibited a slower increase in TVB-N values compared to the CK group, exceeding the standard limit of 15 mg/100 g on day 12 (16.16 mg/100 g) and day 15 (15.85 mg/100 g), respectively. This was likely due to the antimicrobial effect of the chitosan nanoemulsion with perillaldehyde on the beef surface, which retarded protein degradation and reduced formation of amine compounds (Rathod et al., 2024). Furthermore, the nanoemulsion coating on the beef surface acted as an oxygen barrier, inhibiting microbial growth and slowing the protein degradation process, thereby contributing to a lower TVB-N value (Zhang, Liu, et al., 2023). Similarly, Isvand et al. (2024) reported that a where chitosan coating containing lemon essential oil effectively reduced the TVB-N levels in beef during refrigeration. Moreover, Wang et al. (2022a) demonstrated that incorporating chitosan nanoemulsions with thymol effectively slowed the increase in the TVB-N values. Therefore, the present findings suggest that chitosan nanoemulsions loaded with perillaldehyde is an effective strategy for reducing of TVB-N levels in refrigerated beef.

### 3.7 Change in texture

Meat texture is a crucial factor in consumer selection, providing a reliable indication of quality and freshness (Jiang et al., 2024). As illustrated in Table 2, the hardness, springiness, cohesiveness, and chewiness of beef in all groups exhibited a gradual downward trend throughout the refrigeration. Compared to the initial stage of storage, the hardness of CK, CS, and PA-CS groups on day 15 decreased by 75.39 %, 68.34 %, and 55.81 %, respectively, indicating that the PA-CS group better-maintained beef hardness during refrigeration. This could be attributed to the ability of chitosan and perillaldehyde to inhibit the MP oxidation (Fig. 1D), thereby preserving the spatial structure of the proteins and preventing softening of the muscle tissue (Hou et al., 2023). In addition, on day 15, the springiness and cohesiveness of the CK group decreased by 45 % and 26.42 %, respectively, compared to those in on day 0. However, the CS and PA-CS groups exhibited a slower rate of decline, with the PA-CS group maintaining superior springiness and cohesiveness. This phenomenon could be explained by the fact that the chitosan nanoemulsion with perillaldehyde coating enhanced water retention of the beef, thereby reducing water loss. Throughout refrigeration, CS and PA-CS groups consistently exhibited higher chewiness than the CK group, possibly due to their ability to delay MP degradation by inhibiting microbial growth and reproduction (Fig. 4A), and the activity of endogenous enzymes (Ghani et al., 2018). Consequently, the chitosan nanoemulsion incorporating perillaldehyde effectively improved beef texture during refrigeration. Previous studies have reported similar findings, indicating that chitosan-flavonoid or cinnamon essential oil nanoemulsion coatings can help maintain the texture quality of chilled beef (Ghani et al., 2018; Yan et al., 2024).

**Table 2:** Enumeration of texture of beef with or without treatment during refrigeration.

	Storage time (days)					
	0	3	6	9	12	15
Hardness (kg)						
CK	14.39 ± 0.54^Aa^	11.88 ± 0.53^Bb^	8.32 ± 0.62^Cb^	5.74 ± 0.61^Db^	3.98 ± 0.44^Ea^	3.54 ± 0.73^Ea^
CS	14.91 ± 0.31^Aa^	12.96 ± 0.48^Bb^	10.44 ± 0.34^Cab^	8.79 ± 0.65^Da^	6.31 ± 0.54^Eb^	5.72 ± 0.45^Ea^
PA-CS	15.34 ± 0.53^Aa^	14.67 ± 0.49^Aa^	12.57 ± 0.09^Ba^	10.05 ± 0.39^Cab^	7.47 ± 0.59^Db^	6.78 ± 0.38^Da^
Springiness (mm)						
CK	0.8 ± 0.06^Aa^	0.76 ± 0.04^Aa^	0.71 ± 0.03^Aa^	0.62 ± 0.04^Ba^	0.56 ± 0.02^Ca^	0.44 ± 0.04^Da^
CS	0.75 ± 0.07^Aa^	0.74 ± 0.05^Aa^	0.75 ± 0.03^Aa^	0.72 ± 0.02^Aa^	0.61 ± 0.05^Ba^	0.55 ± 0.03^Bb^
PA-CS	0.77 ± 0.04^Aa^	0.76 ± 0.06^Aa^	0.74 ± 0.05^Aa^	0.73 ± 0.02^Aa^	0.67 ± 0.06^ABa^	0.64 ± 0.04^Bb^
Cohesiveness						
CK	0.53 ± 0.04^Aa^	0.58 ± 0.04^Aa^	0.49 ± 0.02^Ba^	0.45 ± 0.03^Bb^	0.41 ± 0.02^Ab^	0.39 ± 0.02^Ab^
CS	0.54 ± 0.02^Aa^	0.53 ± 0.03^Aa^	0.51 ± 0.04^Aa^	0.47 ± 0.02^ABab^	0.43 ± 0.03^Bab^	0.44 ± 0.02^Bab^
PA-CS	0.61 ± 0.05^Aa^	0.58 ± 0.04^Aa^	0.53 ± 0.02^ABa^	0.52 ± 0.03^ABa^	0.47 ± 0.02^Ba^	0.47 ± 0.03^Ba^
Chewiness (N.mm)						
CK	6.42 ± 0.12^Aa^	5.17 ± 0.17^Bb^	3.07 ± 0.19^Cb^	1.7 ± 0.17^Dc^	1.63 ± 0.03^Dc^	1.25 ± 0.15^Ec^
CS	6.41 ± 0.07^Aa^	5.27 ± 0.02^Bb^	3.44 ± 0.2^Cb^	2.89 ± 0.14^Db^	2.25 ± 0.09^Eb^	1.83 ± 0.09^Fb^
PA-CS	6.75 ± 0.11^Aa^	5.71 ± 0.18^Ba^	4.61 ± 0.19^Ca^	3.77 ± 0.08^Da^	3.1 ± 0.14^Ea^	2.58 ± 0.11^Fa^

Note: Values with different capital letter letters (A-F) in the same batch were significantly different (P < 0.05); Values with different lowercase letters (a-c) at the same sampling time were significantly different (P < 0.05).

### 3.8 Change in LF-NMR

LF-NMR was used to assess water migration and distribution in beef during refrigeration. As shown in Fig. 2B, the T_2_ relaxation spectra exhibited three distinct component peaks, representing different water fractions: bound water associated with protein macromolecules and cellular structures (T_2b_), immobilized water confined within the MP structure (T_21_), and free water located in the myofibrillar spaces, contributing to cooking loss (T_22_) (Wu et al., 2021). In the CK group, the relaxation times associated with bound water, immobilized water, and free water increased significantly with storage time. However, CS and PA-CS groups exhibited a slower decline in relaxation time, suggesting that the chitosan nanoemulsion with perillaldehyde helped regulate the water distribution and migration in refrigerated beef. Previous studies have indicated that water migration is closely linked to microbial growth and reproduction (Møller et al., 2010) Accordingly, the antibacterial effects of chitosan and perillaldehyde likely contributed to influencing the water distribution in beef samples. Table 3 presents the T_2_ relaxation time and relative percentages of different T_2_ components over 15 days of storage, indicating that CS and PA-CS groups significantly shortened the relaxation times of T_2b_, T_21_, and T_22_ compared to the CK group as storage time progressed. Furthermore, in CS and PA-CS groups, there was a notable increase in the proportion of immobilized water (PT_21_) and a concurrent reduction in free water (PT_22_), suggesting that the chitosan nanoemulsion with perillaldehyde delayed the conversion of immobilized water into free water. This phenomenon could be attributed to the interaction between the protein, chitosan, and perillaldehyde, which enhanced protein hydration and improved water retention capacity of beef (Sun et al., 2021). Additionally, coating beef with a chitosan nanoelusion was beneficial for reducing water leakage, which aligned with the observed reduction in cooking loss (Fig. 1B). Moreover, the inhibitory effects of chitosan and perillaldehyde on protein denaturation during storage further contributed to the decrease in the proportion of free water. Consequently, the chitosan nanoemulsion with perillaldehyde effectively reduced water loss and improved the quality of beef during refrigeration.

**Table 3:** Enumeration of T_2_ relaxation time and peak area proportion of beef with or without treatment during refrigeration.

	T_2_ relaxation time/ms			P_T2_ (T_2_ peak area proportion)/%		
	T_2b_	T_21_	T_22_	PT_2b_	PT_21_	PT_22_
Day0						
CK	1.17 ± 0.09^Da^	58.01 ± 1.15^Da^	498.91 ± 16.27^Ea^	4.23 ± 0.44^Aa^	94.33 ± 0.30^Aa^	1.44 ± 0.34^Da^
CS	0.96 ± 0.16^Da^	57.45 ± 0.83^Ca^	528.88 ± 25.72^Ca^	4.13 ± 0.21^Aa^	94.24 ± 0.14^Aa^	1.63 ± 0.07^Ba^
PA-CS	0.88 ± 0.06^Da^	58.31 ± 1.29^Ca^	478.91 ± 31.76^Ca^	3.98 ± 0.18^Aa^	94.38 ± 0.42^Aa^	1.64 ± 0.24^Ba^
Day3						
CK	1.15 ± 0.07^Db^	60.22 ± 1.87^Da^	512.36 ± 28.77^Ea^	4.09 ± 0.43^ABa^	94.15 ± 0.41^Aa^	1.76 ± 0.12^Da^
CS	0.99 ± 0.08^Db^	58.5 ± 1.73^Ca^	464.16 ± 34.56^CDa^	4.12 ± 0.56^Aa^	94.26 ± 0.39^Aa^	1.64 ± 0.17^Ba^
PA-CS	0.93 ± 0.12^Da^	59.77 ± 0.34^Ca^	459.54 ± 45.94^Ca^	3.91 ± 0.31^Aa^	94.28 ± 0.69^Aa^	1.82 ± 0.02^Ba^
Day6						
CK	1.12 ± 0.05^Da^	65.98 ± 0.88^Da^	628.87 ± 43.39^Da^	4.18 ± 0.12^Aa^	94.01 ± 0.34^Aa^	1.81 ± 0.27^Da^
CS	0.97 ± 0.09^Da^	60.86 ± 0.79^Cb^	575.48 ± 57.56^Bab^	4.35 ± 0.08^Aa^	94.25 ± 0.15^Aa^	1.40 ± 0.02^Cb^
PA-CS	0.93 ± 0.05^Da^	58.53 ± 1.24^Cb^	519.16 ± 33.68^Ca^	4.09 ± 0.44^Aa^	94.46 ± 0.41^Aa^	1.45 ± 0.04^Cb^
Day9						
CK	1.48 ± 0.08^Ca^	69.67 ± 1.35^Ca^	732.6 ± 48.56^Ca^	3.57 ± 0.2^Bb^	92.96 ± 0.19^Bc^	2.47 ± 0.01^Ca^
CS	1.21 ± 0.14^Cab^	63.53 ± 0.74^Cb^	663.67 ± 35.43^BCa^	4.16 ± 0.18^Aa^	93.97 ± 0.34^Ab^	1.87 ± 0.16^Bb^
PA-CS	1.07 ± 0.04^Cb^	60.46 ± 1.79^BCc^	593.67 ± 20.12^Bb^	4.13 ± 0.34^Aa^	94.34 ± 0.28^Aa^	1.54 ± 0.44^Bb^
Day12						
CK	1.84 ± 0.17^Ba^	75.29 ± 1.07^Ba^	845.48 ± 45.53^Ba^	3.66 ± 0.03^Bb^	92.16 ± 0.07^Cc^	4.18 ± 0.04^Ba^
CS	1.52 ± 0.04^Bb^	67.53 ± 2.24^Bb^	753.63 ± 39.96^Ab^	3.88 ± 0.06^Ba^	93.18 ± 0.33^Bb^	2.94 ± 0.13^Bb^
PA-CS	1.18 ± 0.05^Bc^	63.29 ± 0.98^Bc^	673.12 ± 38.51^ABc^	4.05 ± 0.17^Aa^	94.06 ± 0.17^Aa^	1.89 ± 0.12^Bc^
Day15						
CK	2.39 ± 0.13^Aa^	80.65 ± 1.78^Aa^	979.7 ± 47.54^Aa^	3.19 ± 0.22^Cb^	91.18 ± 0.54^Dc^	5.63 ± 0.12^Aa^
CS	1.81 ± 0.06^Ab^	73.61 ± 2.13^Ab^	807.64 ± 56.52^Ab^	3.57 ± 0.19^Ba^	92.52 ± 0.45^Bb^	3.91 ± 0.14^Ab^
PA-CS	1.56 ± 0.12^Ac^	66.29 ± 1.04^Ac^	723.63 ± 39.96^Ab^	3.82 ± 0.21^Ba^	93.36 ± 0.36^Ba^	2.82 ± 0.16^Ab^

Note: Values with different capital letter letters (A-F) in the same batch were significantly different (P < 0.05); Values with different lowercase letters (a-c) at the same sampling time were significantly different (P < 0.05).

### 3.9 Sensory evaluation

**Fig. 3:**
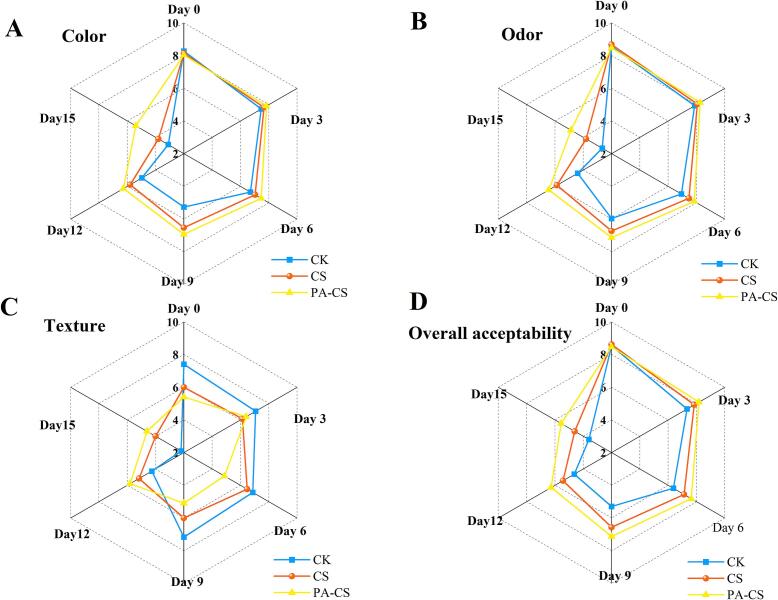
Sensory scores for color (A), odor (B), texture (C), and overall acceptability (D) of beef with and without treatment during refrigeration.

The color, odor, texture, and overall acceptability of refrigerated meat are critical factors influencing consumer purchasing decisions. As displayed in Fig. 3, all sensory attributes of CK and different treatment groups gradually declined over the storage period. However, the CS group exhibited a more rapid decline than the nanoemulsion-treated groups did. By day 9, the CK group reached unacceptable sensory scores (score < 6) for all attributes, whereas CS and PA-CS groups maintained acceptable scores. The decline in sensory quality was closely associated with lipid and protein oxidation. Microbial growth and endogenous enzymatic activity during storage contribute to oxidation, leading to sensory deterioration, including off-flavors, softening, and color deterioration (Ansarian et al., 2022). The higher sensory scores of the PA-CS group throughout storage (except on day 0) could be attributed to the inhibition of spoilage microorganism growth and oxidative processes by chitosan and perillaldehyde. Previous studies have demonstrated that nanoemulsions loaded with natural compounds effectively suppress microbial growth and reduce the formation of oxidation products in stored meat, thereby enhancing sensory attributes (Ansarian et al., 2022; Chaudhary et al., 2022; Snoussi et al., 2022). These findings indicate that the chitosan nanoemulsion with perillaldehyde significantly slowed the deterioration of beef sensory quality, aligning with earlier results on color stability, texture preservation, and TVB-N reduction.

### 3.10 Change in TVC and bacterial community diversity

**Fig. 4:**
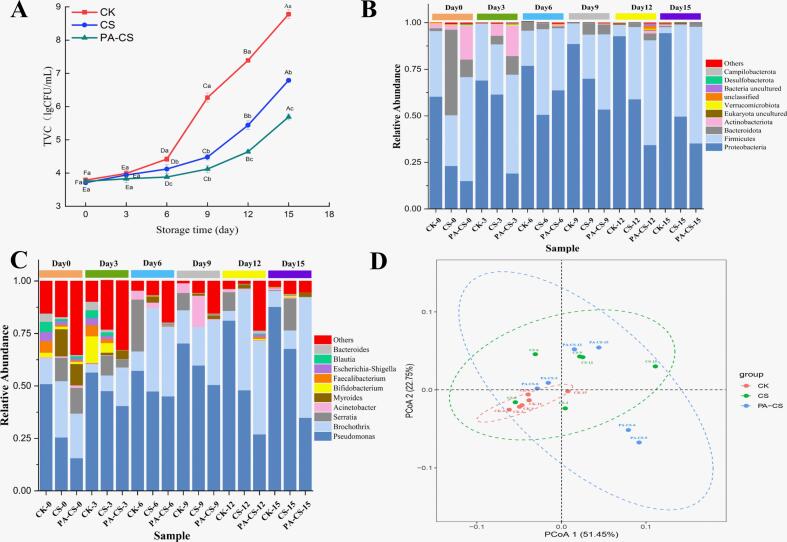
Enumeration of in TVC (A), bacterial communities at the phylum level (B), bacterial communities at the genus level (C), and principal component analysis of bacterial genera (D) of beef with and without treatment during refrigeration. Different capital letters (A-F) indicate significant differences within the same batch (*P* < 0.05); different lowercase letters (a-c) indicate significant differences within the same sampling time (*P* < 0.05).

Microbial spoilage is the primary cause of meat deterioration, with bacterial proliferation playing a crucial role in enzymatic autolysis and oxidative degradation during storage (Rathod et al., 2024). As a result, TVC values are commonly used as indicators of meat freshness during storage. As displayed in Fig. 4A, the TVC values increased significantly in all groups over 15 days of refrigeration, rising from 3.79 to 8.78 CFU/g in the CK group, 3.71 to 6.79 CFU/g in the CS group, and 3.75 to 5.69 CFU/g, in the PA-CS group. The CK group exhibited the highest bacterial growth rate, significantly exceeding that of nanoemulsion-treated groups (*P* < 0.05). According to the National Food Safety Standard for Cooked Meat Products (GB 2726–2016), the acceptable TVC threshold for fresh meat is 4.903 CFU/g. The CK group surpassed this limit on day 9, reaching 6.27 CFU/g, whereas, CS and PA-CS groups remained below the threshold until day 12 (5.44 CFU/g) and day15 (5.69 CFU/g), respectively. These results demonstrate that the chitosan nanoemulsion with perillaldehyde exhibits significant antibacterial effects, effectively prolonging the shelf life of refrigerated beef. This effect can be attributed to the ability of the chitosan nanoemulsion to form a protective coating on the beef surface, which acts as an effective barrier against oxygen, thereby inhibiting microbial growth (Sun et al., 2021). Besides, the strong antimicrobial activity of chitosan and perillaldehyde played a crucial role in the deceased TVC observed during refrigeration. Similar findings have been reported in studies that utilized chitosan nanoemulsions loaded with various natural antibacterial compounds to extend the shelf life of meat products (Chaudhary et al., 2022; Ghaderi-Ghahfarokhi et al., 2017; Sun et al., 2021).

Evaluating bacterial community variations in fresh meat during refrigeration provides insights into the spoilage process, aiding the development of novel meat preservation techniques (Jiang et al., 2024). Fig. 4B depicts the variation in changes community diversity (relative abundance >1 %) at the phylum level across the different nanoemulsion treatments over 15 days of storage. At the initial stage (day 0), the predominant phyla in the CK group were *Proteobacteria, Firmicutes, Bacteroidota,* and *Actinobacteriota.* As storage progressed (day 3, 6, 9, 12, and 15), *Proteobacteria* remained the dominant phylum, with relative abundances of 69.03 %, 76.94 %, 88.06 %, 92.85 %, and 94.53 %, respectively. Previous studies have reported that *Proteobacteria* is the predominant phylum in refrigerated beef (Jiang et al., 2024). *Proteobacteria*, which comprise both aerobic and anaerobic bacteria, are widely recognized as key contributors to meat spoilage. However, in CS and PA-CS groups, the relative abundance of Proteobacteria was significantly lower (49.71 % and 45.25 %, respectively) compared to the CK group. This suggests that chitosan and perillaldehyde effectively inhibited the growth and proliferation of Proteobacteria, thereby delaying the spoilage. This phenomenon could potentially be attributed to the antioxidant and antimicrobial properties of chitosan and perillaldehyde. Erhunmwunsee et al. (2022) found that perillaldehyde is a principal hydrophobic active component present in the essential oil derived from perilla plants, that can effectively inhibit microbial growth. Moreover, the the slow-release effect of perillaldehyde also supplied a sustained inhibition effect on the microbial growth of beef during refrigeration.

Fig. 4C displays the composition and relative abundance of bacterial community diversity (relative abundance >1 %) at the genus level. Ten dominant genera were identified, with *Pseudomonas* (51.65 %) and *Brochothrix* (12.26 %) being the most prevalent in fresh beef storage on day 0 of storage. By day 15, the relative abundance of *Pseudomonas* in the CK group increased significantly to 87.73 %, and it maintained its dominance for the 15 days storage period. *Pseudomonas*, a major cold-tolerant spoilage bacterium with high proteolytic activity, is widely recognized as a key contributor to the decay of meat and meat products, including beef, pork, and chicken. Additionally, Pseudomonas can suppress the growth of other spoilage bacteria through the production of siderophores and sulfur compounds (Guo et al., 2020; Wang et al., 2022; Marcelli, Osimani, & Aquilanti, 2024). Therefore, inhibiting the growth of *Pseudomonas* spp. is crucial for extending the shelf life of fresh meat. The abundance of *Pseudomonas* significantly increased during refrigeration. On day 15, the relative abundance of *Pseudomonas* in the CS and PA-CS groups was significantly lower at 67.73 % and 34.89 %, respectively, compared with 87.73 % in the CK group. This indicates that PA-CS group treatment exhibited superior preservation effects on fresh beef. The enhanced antimicrobial activity could be attributed to the strong antibacterial properties of chitosan and perillaldehyde, along with the prolonged-release capability of the nanoemulsion, which ensured a sustained inhibitory effect on microbial growth. *Brochothrix* is a common facultative anaerobic spoilage bacterium in meat, which not only causes an unpleasant odor in meat but also inhibits the growth of *Pseudomonas*. This also accounted for the decline of *Pseudomonas* (Xu et al., 2024). Notably, although the abundance of *Brochothrix* significantly decrease during refrigeration in CK and CS groups, the abundance of *Brochothrix* in PA-CS group exhibited an increased trend. This phenomenon could be explained that perillaldehyde exhibited a poor inhibitory effect of *Brochothrix* growth and chitosan can act as a rich carbon source for *Brochothrix* growth (Cao et al., 2023). Therefore, the chitosan nanoemulsion and perillaldehyde coating helps to decrease microbial diversity of beef and inhibit the growth of spoilage bacteria such as *Pseudomonas*, resulting in the improvement of beef quality.

Fig. 4D displays the principal coordinate analysis (PCoA) of bacterial communities. PC1 primarily represented the variation in the dominant bacterial species, whereas PC2 reflected the differences in their relative abundance. Together, PC1 and PC2 accounted for 51.45 % and 22.75 % of the total variances, respectively. The CK group was distinctly separated from CS and PA-CS groups, indicating that chitosan nanoemulsion treatment significantly altered the bacterial community structure of fresh beef during the 15 days storage period. Notably, PA-CS treatment had a more pronounced effect on bacterial composition and distribution. These findings suggest that the chitosan nanoemulsion with perillaldehyde not only influenced the relative abundance of predominant bacteria, but also had a greater impact on the composition of the dominant bacterial species.

### 3.11 LEFSe analysis

**Fig. 5:**
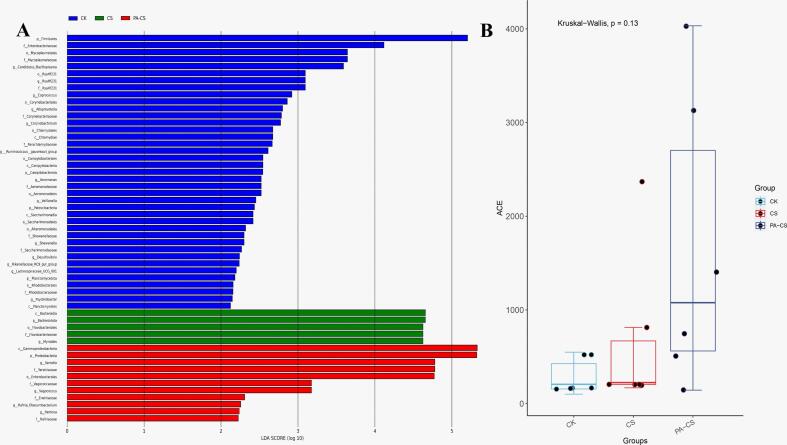
Enumeration of bacterial LEfSe analysis (A) and abundance-based coverage estimator (B) of beef with and without treatment during refrigeration.

As illustrated in Fig. 5A and B, a clear distinction was observed between CK and nanoemulsion-treated groups during 15 days refrigeration period. To further analyze these differences, LEFSe analysis was conducted to identify bacterial community variations among the treatment groups. A longer bar indicated a more substantial impact on the differences between groups. In the CK group, the bacterial community composition was highly diverse during storage. However, CS and PA-CS groups exhibited reduced bacterial diversity, with the PA-CS group exhibiting the lowermost diversity. Notably, the CK group had higher abundances of *Brochothrix*, *Enterobacteriaceae,* and *Mycoplasmatales*, distinguishing it from the nanoemulsion-treated groups. Meanwhile, in the CS group, the dominant bacterial taxa included *Flavobacteriaceae* and *Myroides*, whereas the PA-CS group exhibited significant differences in the abundances of *Gammaproteobacteria* and *Pseudomonadaceae* compared to other groups. This was because the chitosan and perillaldehyde have a good antioxidant activity and antibacterial ability, inhibiting lipid oxidation, protein degradation, and bacterial growth. Therefore, the bacterial diversity and richness in PA-CS group was reduced during refrigeration. Moreover, the barrier effect of chitosan nanoemulsions also play a crucial role in limiting bacterial growth, thereby reducing microbial diversity of beef. Thus, the PA and PA-CS groups can act as a barrier between the beef and the external environment, inhibiting bacterial growth, and slowed down the lipid and protein oxidation, contributing to the shelf-life extension of meat and meat products.

### 3.12 Pearson correlations

**Fig. 6:**
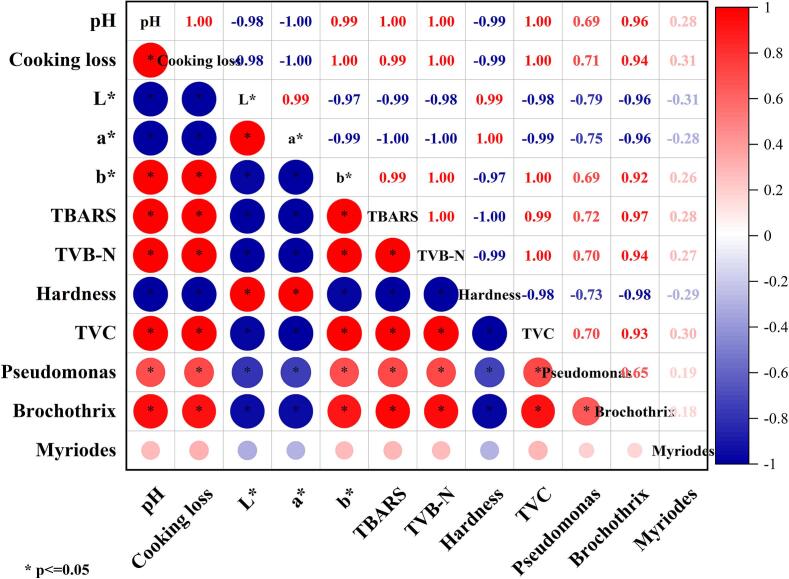
Pearson correlation analysis of quality properties and microorganism genus levels in the PA-CS group.

The existing correlations between the physiochemical, microbial, and texture characteristics in beef samples of PA-CS group were preliminarily analyzed and depicted in Fig. 6. The results showed that the chitosan nanoemulsion loading with perillaldehyde can effectively influence the bacteria that improve the beef quality during refrigeration. The presence of *Pseudomonas and Brochothrix* positively correlated with pH, Cooking loss, TBARS, TVB-N and *b*^*⁎*^, of beef, whereas negatively correlated with *L*^*⁎*^, *a*^*⁎*^ and hardness (*P* < 0.05). Previous researches have demonstrated that *Pseudomonas* can secreted plenty of proteolytic lipolytic enzymes in connection with protein degradation and lipid oxidation, producing alkaline metabolites and contributing to the increasing pH and TVB-N of refrigerated beef (Cao et al., 2023). In addition, Bro*chothrix* also may decompose protein and lipid, leading to the production of associated with off-odors, such as 3-hydroxy-2-butanone (acetoin), 2,3-butanedione (diacetyl), and 3-methyl-1-butanol (Genotypic and phenotypic characterization of the food spoilage bacterium *Brochothrix thermosphacta*). Thus, understanding the bacterial changes during beef storage is crucial for developing quality control and preservation strategies for refrigerated beef. Moreover, Fig. 6 also demonstrated the significant positive correlation among cooking loss, pH, b^⁎^, TVC, TBARS and TVB-N. Hardness, *L*^*⁎*^, *a*^*⁎*^ shown negative correlations with other physiochemical and microbial characteristics. On the basis of Pearson correlations analysis, physiochemical, microbial, and texture indictors exhibited a significant correlation. Thus, a comprehensive understanding of quality correlation of beef during refrigeration can facilitate the application of PA-CS coatings to enhance the quality and extend the shelf life of various fresh meats.

## 4 Conclusion

In this study, the PA-CS coating were successfully developed into edible packaging materials, which can improve the cooking loss, texture, and water migration and reduce TVC, oxidation, and color deterioration of fresh beef during refrigeration. The PA-CS coating exert a barrier effect with good antioxidant activity and antibacterial ability, inhibiting lipid oxidation, protein degradation, and bacterial growth. Meanwhile, the PA-CS coating also facilitated slowing down the release of the active compounds on the surface of pork samples, thereby extending their effective duration of action. In addition, a more in-depth and comprehensive understanding of the relationship between meat quality and microbial diversity in the preservation of refrigerated beef was achieved. Overall, these findings suggest that PA-CS coating have great potential in acting a novel active packaging for fresh beef preservation. Nevertheless, in future research, greater emphasis should be placed on investigating how the incorporation of essential oils affects the sensory attributes of the coatings, as these changes could significantly impact consumer acceptance of fresh meat products. Moreover, the safety of perillaldehyde must also be verified through comprehensive testing, which will necessitate the use of both laboratory and in vivo methods, contributing for better applications in food preservation.

## CRediT authorship contribution statement

**Shengming Zhao:** Writing – review & editing, Writing – original draft. **Jingyao Wu:** Software, Methodology. **Mengke Li:** Methodology, Data curation. **Yanyan Zhao:** Investigation, Conceptualization. **Guoyuan Xiong:** Supervision, Resources, Funding acquisition. **Xinkun Wang:** Writing – review & editing, Visualization. **Lizeng Peng:** Project administration, Funding acquisition.

## Ethical guidelines

Ethical permission was granted by our institution.

## Declaration of competing interest

The authors declare that they have no known competing financial interests or personal relationships that could have appeared to influence the work reported in this paper.

## Data Availability

Data will be made available on request.

## Appendix A Supplementary data

Certificate of English Editing Image 1
