# Supplementary material for: Assessment of chitosan nanoemulsion loading with perillaldehyde on the quality characteristics and microbial diversity of beef slices during refrigeration storage
Source: Food Chem X. 2025 Jun 12;29:102658. doi: 10.1016/j.fochx.2025.102658 (PMC12213107; doi:10.1016/j.fochx.2025.102658)
Supplement: Supplementary file 1 — Certificate of English Editing [file mmc1.pdf]

Freescience Editorial Team

Certificate of English Editing

Paper Title

Assessment of chitosan nanoemulsion loading with perillaldehyde on the quality characteristics and microbial diversity of beef slices during refrigeration storage

Authors

Shengming Zhao, Jingyao Wu, Mengke Li, Yanyan Zhao, Guoyuan Xiong, Xinkun Wang, Lizeng Peng

This certificate is issued as a confirmation that the paper mentioned above has been proofread and edited for language clarity and grammar by professional editors of our company.

We guarantee that the original message was not distorted, and that the paper is understandable and free of errors assuming that the changes and suggestions given are accepted, and text is not altered without our knowledge.

Date of Editing: 04-02-2025
